# Trends in Suicide Mortality by Method from 1979 to 2016 in Japan

**DOI:** 10.3390/ijerph16101794

**Published:** 2019-05-21

**Authors:** Bibha Dhungel, Maaya Kita Sugai, Stuart Gilmour

**Affiliations:** 1 Graduate School of Public Health, St. Luke’s International University, Tokyo 104-0045, Japan; 18mp201@slcn.ac.jp; 2 Department of Global Health Policy, Graduate School of Medicine, The University of Tokyo, Tokyo 113-0033, Japan; maayakitasugai@gmail.com; 3 Institute of Global Health Policy Research, Bureau of International Health Cooperation, National Center for Global Health and Medicine, Tokyo 162-8655, Japan

**Keywords:** suicide, Japan, injury, Poisson regression, mortality

## Abstract

Suicide is a major public health concern in Japan. This study aimed to characterize the trends in suicide mortality in Japan by method since 1979. Using data from the Japan vital registration system, we calculated age-standardized rates of suicide mortality separately by sex and method. We conducted a log-linear regression of suicide mortality rates separately by sex, and linear regression analysis of the proportion of deaths due to hanging, including a test for change in level and trend in 1998. While crude suicide rates were static over the time period, age-adjusted rates declined. The significant increase in suicide mortality in 1998 was primarily driven by large changes in the rate of hanging, with suicide deaths after 1998 having 36.7% higher odds of being due to hanging for men (95% CI: 16.3–60.8%), and 21.9% higher odds of being due to hanging for women (95% CI: 9.2–35.9%). Hanging has become an increasingly important method for committing suicide over the past 40 years, and although suicide rates have been declining continuously over this time, more effort is needed to prevent hanging and address the potential cultural drivers of suicide if the rate is to continue to decline in the future.

## 1. Introduction

Suicide is a major public health concern. In 2016, around 800,000 suicide deaths occurred worldwide, with an annual global age-standardized suicide rate of 10.53 per 100,000 [1]. Globally, suicide is the second-leading cause of death among people aged 15–29 years, and it accounts for about 1.4% of premature deaths. Histories of medical disorders such as depression, mood disorder, and cognitive impairment have been identified as important risk factors for suicide [2], but sociocultural factors are also likely to play a major role in suicide epidemiology. There are significant sex imbalances in risk, with a ratio of three male deaths for every female death in high-income countries, and three male deaths for every two female deaths in low-income and middle-income countries. The global age-standardized suicide rate fell 26% (23% in men and 32% in women) from 2000 to 2012, but this pattern varies by country or region [3]. Little is known about the reason for large spatial and temporal variations in suicide rates. 

Japan has long battled one of the highest suicide rates in the industrialized world [4]. Japan ranked 14th for suicide rates globally in 2018, and suicide was the sixth leading cause of death in Japan in 2015 [5,6]. Hanging has been consistently recorded as the most frequently used suicide method, and makes a major contribution to the overall suicide mortality rate, especially among people aged 50 years and above [7,8,9,10]. As the dominant method of suicide, hanging accounts for more than 60% of suicide deaths in Japan [11,12,13]. Hanging has been shown to be one of the most lethal suicide methods, with a fatality rate of over 83% [14,15], and small changes in the distribution of methods of suicide can have the potential to drive large changes in the overall rate [16], making action on restricting access to certain methods of hanging a common public health strategy for reducing suicide mortality [17,18,19].

From 1960 until 1995, the crude suicide mortality rate in Japan decreased from 27.7 [20] to 17.1 per 100,000 before increasing suddenly in 1998 to a rate of 25.3 per 100,000. Previous studies have reported that this increase may be closely related to unemployment and economic hardship during the same period [21]. However, it is possible that these changes reflect aging or changes in the distribution of methods of suicide. In Japan, the suicide rate increases with the increase in age, with rates among men twice those of women [22]. Previous research on suicide typically analyzed crude suicide rates [23], which are influenced by the age composition of the population [24,25]. Since the age distribution of Japan has changed rapidly over time, the use of crude, rather than age-adjusted, rates in the measurement of suicide trends could be misleading. 

In this study, we analyzed patterns of suicide by sex, age, and method to discover the relationship between these risk factors and describe the specific features of suicidal behavior in Japan [22]. 

The objectives of the research are to:

1. Characterize the trends in and risk factors for suicide in Japan
2. Assess the role of aging in the increase in suicide rates in 1998
3. Identify the magnitude of the increase in 1998 and the extent to which it is driven by changes in method

Analyzing trends in method-specific suicides may help to understand the background of recent trends in overall suicide rates in countries such as Japan where rapid changes have been observed, especially in high-income countries, which face a high burden of suicide-related mortality in a rapidly aging population. It may also help to explain the swift changes that have been observed in Japan during the 1990s, and shed some light on similar rapid changes that occurred at this time in other developed nations such as Australia [26]. 

## 2. Materials and Methods

Data on suicide mortality were obtained from the vital statistics registration of the Ministry of Health, Labor, and Welfare (MHLW) in Japan [6]. This database provides complete coverage of all the deaths that have occurred in Japan with the cause of death coded using the International Classification of Disease (ICD)-9 or ICD-10 codes, and is available upon the submission of an application by researchers based in Japan and holding appropriate authority. For an analysis of overall trends, we computed age-adjusted directly standardized suicide rates with the 2010 population of Japan as the standard population [27]. In this research, we divided age into four groups (15–29, 30–59, 60–79, and 80+ years) and compared the trends in suicide by age group and method. 

To analyze trends in suicide rates by gender, age, and methods used between 1979–2016, we performed Poisson regression analysis. We entered age group, suicide method, year, and a variable indicating whether the suicide occurred before or after 1998 to capture the sudden rise occurring in this year. Then, we included interactions for year, age category, sudden rise, and method of suicide to estimate trends in different methods by age category before and after 1998 as well as to determine the percent change in suicide rates (change in level) after 1998. A linear regression analysis was conducted by sex to estimate changes in the proportion of suicide deaths that were due to hanging. Details of the model interpretation with specific terms are given in Supplementary file. 

We excluded children aged under 15 years from the regression analysis due to the very low suicide rate in this age group. We used different models for men and women due to the variation in patterns as well as choice of methods. Linear combinations of the key variables (year, suicide method, age category, 1998 increase) were calculated in order to estimate the change in suicide rate before and after 1998 separately for age and suicide category with 95% confidence intervals. These linear combinations were presented as annual percentage changes (for trends) or absolute percentage changes (for the change in level in 1998). We conducted all analyses in Stata/IC version 15 (Stata Corp LP, College Station, TX, USA).

## 3. Results

### Trends in and Risk Factors for Suicide in Japan

**Figure 1:**
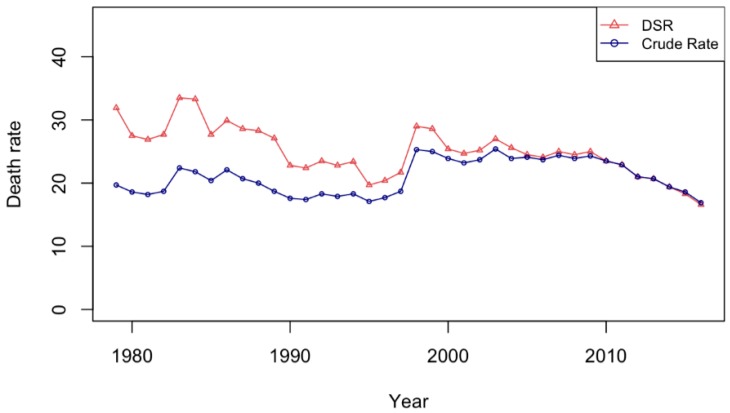
Crude and standardized suicide rates from 1979 to 2016.

Figure 1 shows the age-standardized and crude suicide rates for the period 1979 to 2016. It is clear that after age standardization, the trend in suicide was decreasing until 1995; this was in contrast to the trend in crude rates, which were stable between 1979–1995. The sudden rise in 1998 did not change the broad downward trend in suicide.

**Figure 2:**
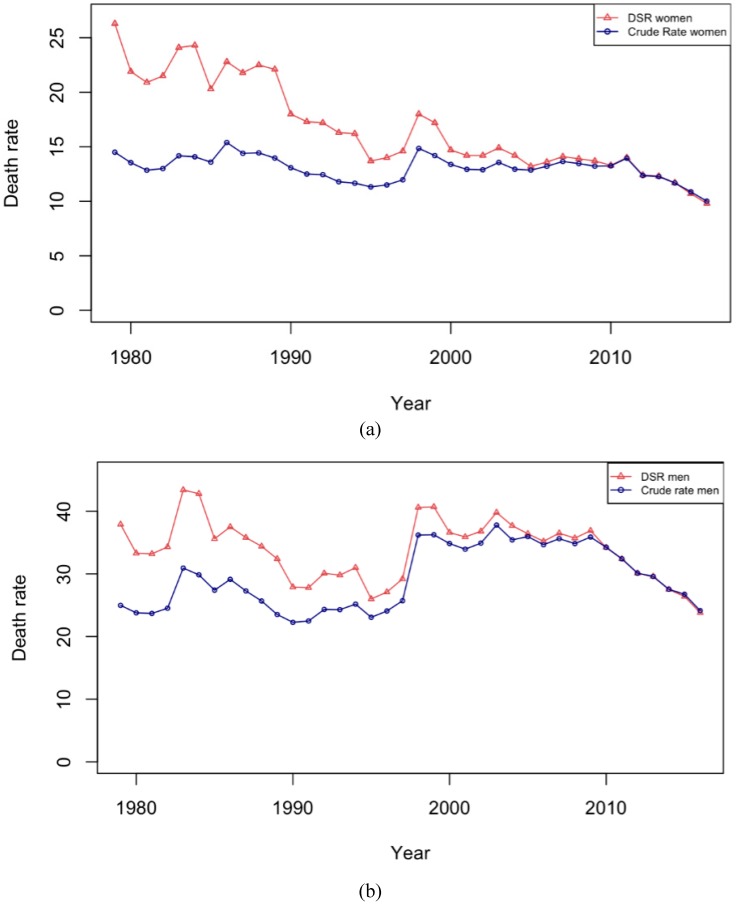
Crude and standardized suicide rates by sex: (**a**) men, (**b**) women.

Figure 2 shows the age-standardized and crude suicide rates by gender. From the figure, it is evident that the suicide rate among men is higher than that among women. For men and women, the age-adjusted rate was decreasing between 1979–1997, rose sharply in 1998, and then declined steadily, whereas the crude rates plateaued for about 10 years after reaching their highest level in 1998. This suggests that crude rates in Japan over the past 30 years have been heavily affected by the aging of the population, which drives a growing proportion of the population into higher-risk age groups.

**Figure 3:**
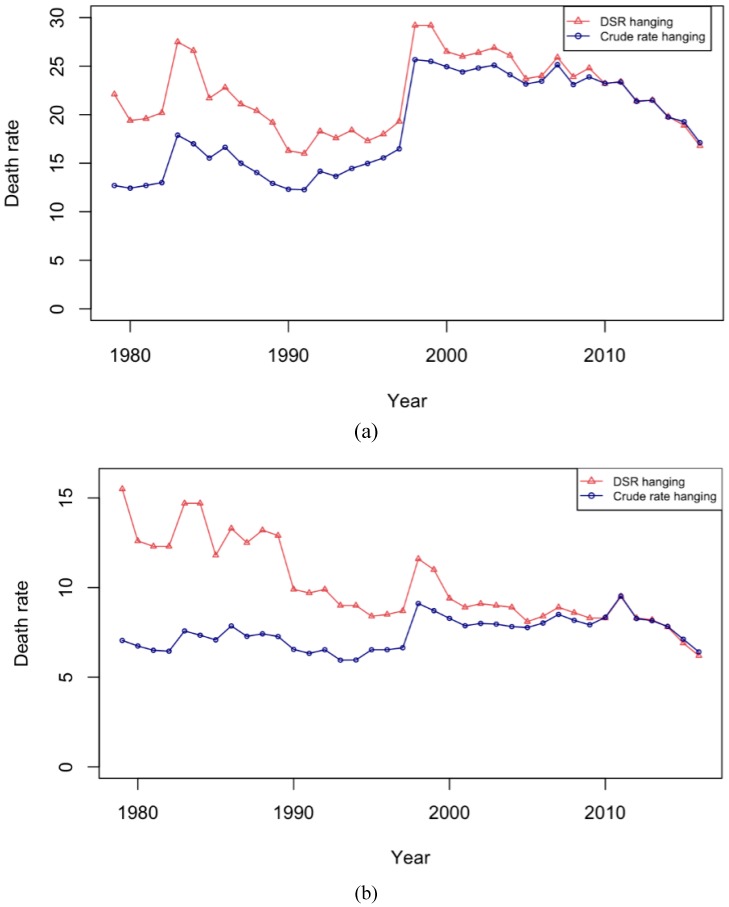
Crude and standardized suicide rates due to hanging among (**a**) men and (**b**) women.

One of the most lethal methods of suicide, hanging, is the most frequently used method and contributes to a large proportion of the overall suicide rates in Japan, and deserves special investigation. Thus, the age-adjusted and crude suicide rates due to hanging for men and women respectively are depicted in Figure 3. The rate of suicide mortality by hanging was higher in men compared to women. In the late 1990s, suicide by hanging increased sharply for both men and women of all age groups. Even for hanging, the trend in the age-standardized suicide rate was decreasing until 1998, when it peaked, and then continued to decline again among men. In the case of women, the rise in age-standardized suicide rates in 1998 slowed down the decreasing trend. Hanging-related suicide in women remained low from the year 2000 onwards. The graph of overall suicide trends (Figure 1) closely resembles the graph of suicide by hanging among men and women (Figure 3), indicating that suicide rates in Japan are mostly driven by hanging.

**Figure 4:**
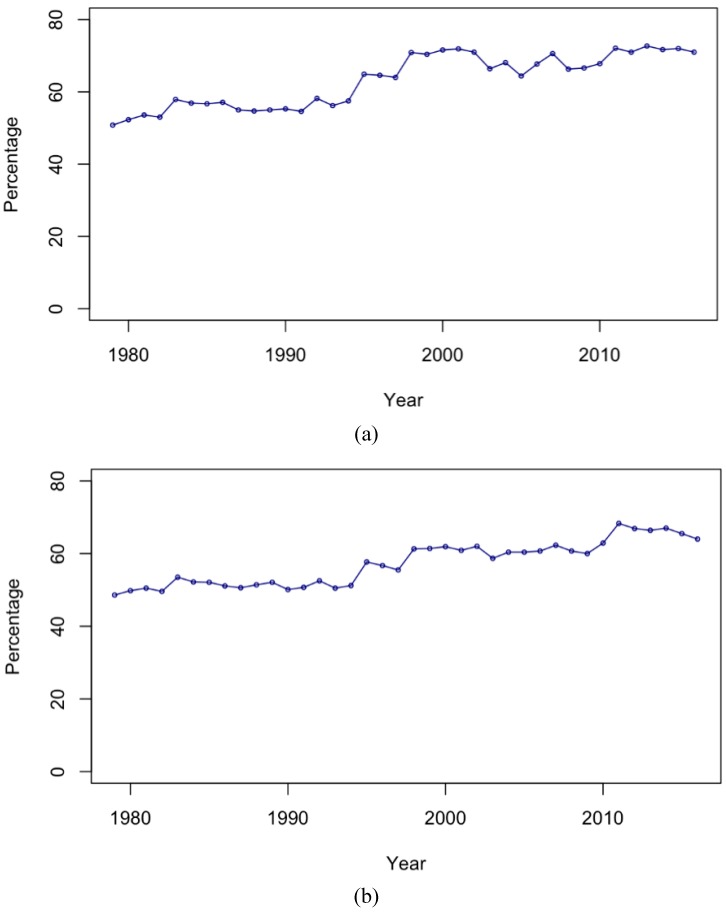
Proportion due to hanging for (**a**) men and (**b**) women.

Figure 4 shows the proportion of all suicide deaths that were due to hanging, by gender, over the time period. It clearly shows that hanging was the most commonly used method to commit suicide. It consistently contributed to more than 50% of the suicide deaths from 1979 until 2016 among both men and women, and the proportion has been growing over time. Table 1 shows the results of a linear regression of the proportion of deaths that are due to suicide, including a term for the 1998 increase. There was an annual 1.3% statistically significant increase in the odds that a suicide death would be due to hanging for both sexes. In 1998, when there was a sharp increase in the crude and standardized suicide rates and rates due to hanging, there was a statistically significant increase in the odds that a death would be due to hanging, by 36.7% among men and 21.9% among women.

Full results of the Poisson regression of suicide rates by age, method, and before and after 1998 are shown in Supplementary file. Table 2 summarizes the suicide trends before and after 1998, with coefficients presented as incidence rate ratios. Among men, suicide by hanging was increasing for the 15–29 age group after 1998, while it was decreasing for all the other age groups. Suicide by gas was increasing in most male age groups after 1998, and this increase was large compared to all the other suicide categories. For women, suicide by hanging was increasing slightly after 1998 in all the groups except for those aged above 80. However, the annual trend in suicide by gas among women was increasing steadily after 1998. Table 2 shows that rates of hanging-related suicide were declining in older age groups before 1998, and continued to decline after 1998. 

Table 3 shows the level change in suicide that occurred in 1998 by age and suicide method. For men, suicide by hanging increased drastically among the 30–59 and 60–79 age groups. Similarly, suicide by gas rose among the age group 30–59, while it declined in large percentages among the 15–29 and above-80 age groups. Suicide by poisoning among men increased significantly in 1998. In women, the change in level of hanging suicide was high in the oldest age group. Despite these large increases in non-hanging-related suicides in 1998, the large increase in hanging in those aged 30–79 dominated the overall profile of suicide in Japan, so that after 1998, the proportion of suicides due to hanging was greater than 60% in both men and women (Figure 4).

## 4. Discussion

This study found broad changes in the trends in suicide after adjusting for age. The age-adjusted suicide trend was decreasing in Japan both before and after 1998. The apparent sudden increase in 1998 had no effect on this downward trend. This shows that the apparent high rates of suicide observed in previous studies are a consequence of aging. Hanging was the most commonly used method to commit suicide among both men and women throughout the study period. Although the non-hanging-related suicide rate increased in 1998, the large increase in hanging among people aged 30–79 dominated the overall increase. Furthermore, while a large increase in overall suicide rates occurred in 1998, it was almost entirely driven by a large increase in rates of hanging, suggesting causal factors other than the economic crisis of the 1990s. After 1998, the proportion of suicides due to hanging remained above 60% throughout the study period, and continued to increase. The sudden increase in hanging at this time suggests a social contagion or some other cultural effect, and more research is needed in order to understand what factors affected suicide at this time in Japan.

This is the first study to our knowledge to analyze Japanese suicide data by adjusting for age separately by gender and method, and measure the level change in suicide in 1998 using Poisson regression analysis. Although previous studies have analyzed method-specific suicide trends [9,10], some of these studies did not adjust for aging or model separately by sex. Other studies used joinpoint analysis to identify the increased rates in 1998 and the large contribution to this increase due to hanging, but had a limited range of data in the pre-1998 comparison period, and did not perform a difference-in-difference analysis [10,28]. Our study helps understand the contribution of age-standardized method-specific data to the sharp shifts in the national suicide rates. Previous studies have identified a sudden increase in suicide rates in 1998 and a long-term stable rate of suicide, suggesting that economic stress may be responsible for the 1998 increase [29,30]. Factors such as work-related stress, family or partner issues, and economic stress are known to be major risk factors for suicide in Japan [31]. However, our results show that suicide rates have been declining despite the stable presence of these risk factors over the past 30 years, and suggest that recent government prevention efforts have been effective. The only significant increase we observed over the past 30 years was the sudden increase in rates in 1998. It has been previously shown that the largest increase in 1998 compared with the three-year average between 1995–1997 was observed among older men who were unemployed, self-employed, or in managerial positions [31]. However, our study has shown that after adjusting for the aging of the Japanese population over this period, suicide rates are declining, and the 1998 increase was most likely due to sudden increases in only one method of suicide: hanging. This suggests that previous findings on suicide and its causes in Japan were incorrect, and that there are social or cultural factors underlying the 1998 rise that may reflect changes in method. As an example, suicide-related internet use such as suicide bulletin board systems in Japan first began to occur in the mid-1990s, with people gathering on suicide-related bulletin board systems (BBSs) at this time [32]. The use of these BBSs is known to have an adverse effect on the mental health of young and middle-aged people [33,34]. The growth of these BBSs and other media depictions of suicide at this time may have had some causative effect on suicide rates, indicating a cultural change or cultural contagion rather than a socioeconomic cause. In addition to the growth of suicide-related BBSs, the publication of bestselling guides to suicide that ranked certain methods [35] indicate a cultural phenomenon of increased attention to suicide during this period, and this change in the popular awareness of suicide—and of particular methods—may have some association with the large increase in hanging that was observed at this time. More research is needed on the specific cultural drivers of suicide in Japan before definitive conclusions can be drawn about the causes of the late-1990s suicide peak, but it is possible that the timing of this increase and the large increase in hanging may indicate that this phenomenon was not directly related to economic changes at this time.

This study has several limitations. No covariates other than age and gender were used for confounder adjustment. The data were not disaggregated by prefecture or municipality, which might provide more detailed information on the role of hanging and aging in the change in level. Transition in the ICD version from ICD-9 to ICD-10 over the period studied [36] could have impacted estimates of suicide arising from changes in disease coding. The reliability of suicide statistics could underestimate total reported suicide deaths [37], which may have had an effect on our results, although its extent could not be evaluated due to an absence of evidence. We also did not consider an age–period–cohort model, which might further explain the role of hanging in the overall increase in suicide [38].

A common approach for reducing suicide is limiting the access to the commonly used methods [39]. Previous works have shown that restriction of firearms [40,41], pesticides [42], and domestic gas [43] led to decreases in method-specific suicide rates. However, this approach is not suitable for suicides by hanging, as the ligature that is commonly used is easily available. Nonetheless, there are studies suggesting that restriction to access does not reduce suicide rates, but instead stimulates the individual to shift to alternative methods of suicide [44,45,46,47,48]. Among the various factors that contribute to the choice of a suicide method, the social acceptability of the method as well as the cultures, traditions, and values attached to it play a major role as well [49,50]. In other high-income Asian countries, the pattern of suicide methods is different to that of Japan; for example, jumping from a height is the most common cause of death in Singapore [51]. This may be due to differences in the built environment between these communities, or due to cultural and religious factors affecting the choice of method, for example due to a desire to avoid disfigurement of the body, or due to different suicide prevention strategies already in place in different countries. As the proportion of suicide due to hanging has been changing over time in Japan, further research on the cultural and social factors affecting suicide is important, especially in relation to hanging. The correct understanding of suicide trends based on age structure in an aging country such as Japan, and identifying specific changes in suicide methods is essential for effective suicide prevention in the future. Nonetheless, the general downward trend in age-specific suicide rates in this study suggests that progress is being made on suicide-related mortality in Japan. However, further interventions in the 30–79 age group are necessary in order to make further gains in reducing the major causes of suicide mortality in Japan. Public health planners and policymakers should consider specific actions to reduce the growing appeal of hanging as a method of suicide in Japan by identifying the social drivers of this method in particular (including, potentially, internet-based media supporting suicide, group suicides, and other social contagions) and acting to counter them. The government should consider media guidelines similar to those introduced in Australia, which restrict the reporting of suicide and give particular advice about how to report suicide, including the provision of details about suicide help lines in all reports of suicide. In the Japanese context, these guidelines should extend beyond news reports to a new code of conduct for the representation of suicide in movies, popular television, and manga. Hanging is the most difficult suicide method to prevent, but by taking these actions, the government can reverse the apparently inexorable growth of this method.

## 5. Conclusions

The increasing rates of suicide in Japan reflect the super-aging Japanese society, although the suicide trend is declining in all of the most-affected age groups. The majority of the suicide deaths in the past 38 years was driven by hanging, and the proportion has been growing over time. With the progress that has been made in the prevention of suicide, rates are declining. Increased attention on finding effective intervention strategies that are particularly aimed at hanging and in people aged 30–79 could work to further drive these rates down, and enable Japan to maintain the progress that it has made in fighting this tragic and preventable cause of death over the past 30 years.

## Figures and Tables

**Table 1:** Odds ratio of proportion of suicide deaths due to hanging by sex, 1979–2016.

Variables	Odds Ratio	95% CI	*p*-Value
**Men**			
Year	1.013	(1.006–1.021)	<0.01
1998 increase	1.367	(1.163–1.608)	<0.01
**Women**			
Year	1.013	(1.008–1.018)	<0.01
1998 increase	1.219	(1.092–1.359)	<0.01

Note. CI = Confidence Interval.

**Table 2:** Suicide trends before and after 1998.

Suicide Category	Before 1998		After 1998	
	IRR	95% CI	IRR	95% CI
		**Men**		
**Age 15–29**				
Hanging	0.989	(0.986–0.991)	1.012	(1.010–1.015)
Gas	0.901	(0.897–0.906)	1.075	(1.070–1.080)
Drowning	0.961	(0.951–0.970)	0.981	(0.967–0.995)
Poisoning	0.950	(0.942–0.959)	0.955	(0.944–0.966)
Others	0.988	(0.985–0.991)	0.981	(0.977–0.985)
**Age 30–59**				
Hanging	0.998	(0.997–1.000)	0.975	(0.974–0.976)
Gas	0.957	(0.954–0.959)	1.005	(1.003–1.007)
Drowning	0.988	(0.983–0.992)	0.935	(0.929–0.940)
Poisoning	0.962	(0.959–0.966)	0.942	(0.938–0.947)
Others	0.993	(0.991–0.995)	0.962	(0.960–0.964)
**Age 60–79**				
Hanging	0.987	(0.985–0.988)	0.971	(0.970–0.972)
Gas	0.998	(0.989–1.006)	0.984	(0.979–0.989)
Drowning	0.968	(0.961–0.975)	0.968	(0.961–0.974)
Poisoning	0.985	(0.980–0.990)	0.915	(0.909–0.921)
Others	1.001	(0.997–1.005)	0.966	(0.963–0.969)
**Age 80+**				
Hanging	0.961	(0.958–0.964)	0.967	(0.965–0.970)
Gas	0.896	(0.864–0.930)	1.015	(0.990–1.041)
Drowning	0.921	(0.907–0.934)	0.955	(0.940–0.970)
Poisoning	0.984	(0.970–0.997)	0.943	(0.932–0.954)
Others	0.946	(0.938–0.954)	0.958	(0.951–0.965)
		**Women**		
**Age 15–29**				
Hanging	0.989	(0.984–0.993)	1.015	(1.012–1.019)
Gas	0.865	(0.857–0.873)	1.087	(1.077–1.096)
Drowning	0.929	(0.918–0.940)	0.973	(0.957–0.990)
Poisoning	0.950	(0.941–0.959)	0.993	(0.983–1.002)
Others	0.996	(0.993–1.000)	0.971	(0.966–0.975)
**Age 30–59**				
Hanging	0.988	(0.986–0.990)	1.001	(0.999–1.003)
Gas	0.905	(0.899–0.911)	1.038	(1.033 –1.043)
Drowning	0.976	(0.971–0.980)	0.935	(0.929–0.941)
Poisoning	0.959	(0.954–0.963)	0.976	(0.971–0.982)
Others	1.005	(1.002–1.008)	0.979	(0.977–0.982)
**Age 60–79**				
Hanging	0.965	(0.963–0.967)	0.974	(0.973–0.976)
Gas	0.883	(0.870–0.897)	1.017	(1.005–1.030)
Drowning	0.949	(0.944–0.953)	0.964	(0.959–0.969)
Poisoning	0.983	(0.978–0.988)	0.924	(0.918–0.930)
Others	0.993	(0.989–0.998)	0.976	(0.972–0.980)
**Age 80+**				
Hanging	0.962	(0.959–0.965)	0.944	(0.942–0.947)
Gas	0.884	(0.845–0.924)	1.002	(0.965–1.040)
Drowning	0.941	(0.934–0.948)	0.923	(0.914–0.931)
Poisoning	1.004	(0.992–1.016)	0.928	(0.918–0.938)
Others	0.969	(0.959–0.979)	0.952	(0.943–0.960)

Note. IRR = Incidence rate ratio; CI = Confidence interval.

**Table 3:** Level change in suicide at 1998.

Suicide Category	IRR	95% CI	Percent Change	95% CI
**Men**				
**Age 15–29**				
Hanging	1.154	(1.083–1.229)	15.4	(8.3–22.9)
Gas	0.099	(0.087–0.113)	−90.1	(−91.3–−88.7)
Drowning	0.711	(0.485–1.042)	−28.9	(−51.5–−4.2)
Poisoning	1.725	(1.279–2.325)	72.5	(27.9–132.5)
Others	1.268	(1.144–1.405)	26.8	(14.4–40.5)
**Age 30–59**				
Hanging	2.957	(2.875–3.042)	195.7	(187.5–204.2)
Gas	1.004	(0.945–1.067)	0.4	(−5.5–6.7)
Drowning	3.310	(2.826–3.877)	231.0	(182.6–287.7)
Poisoning	1.811	(1.583–2.072)	81.1	(58.3–107.2)
Others	2.301	(2.170–2.439)	130.1	(117.0–143.9)
**Age 60–79**				
Hanging	2.221	(2.136–2.309)	122.1	(113.6–130.9)
Gas	2.891	(2.463–3.393)	189.1	(146.3–239.3)
Drowning	1.308	(1.071–1.597)	30.8	(7.1–59.7)
Poisoning	3.911	(3.289–4.650)	291.1	(228.9–365.0)
Others	2.384	(2.167–2.622)	138.4	(116.7–162.2)
**Age 80+**				
Hanging	1.109	(1.021–1.203)	10.9	(2.1–20.3)
Gas	0.238	(0.108–0.526)	−76.2	(−89.2–−47.4)
Drowning	0.607	(0.388–0.951)	−39.3	(−61.2–−4.9)
Poisoning	2.267	(1.604–3.204)	126.7	(60.4–220.4)
Others	0.987	(0.783–1.245)	−1.3	(−21.7–−24.5)
**Women**				
**Age 15–29**				
Hanging	1.277	(1.146–1.422)	27.7	(14.6–42.2)
Gas	0.044	(0.034–0.058)	−95.6	(−96.6–−94.2)
Drowning	0.627	(0.397–0.988)	−37.3	(−60.3–−1.2)
Poisoning	1.002	(0.767–1.308)	0.2	(−23.3–30.8)
Others	1.804	(1.586–2.052)	80.4	(58.6–105.2)
**Age 30–59**				
Hanging	1.132	(1.071–1.196)	13.2	(7.1–19.5)
Gas	0.237	(0.203–0.277)	−76.3	(−79.7–−72.3)
Drowning	2.327	(1.970–2.747)	132.7	(97.0–174.7)
Poisoning	0.836	(0.713–0.980)	−16.4	(−28.7–−2.0)
Others	1.748	(1.613–1.894)	74.8	(61.3–89.4)
**Age 60–79**				
Hanging	1.079	(1.021–1.140)	7.9	(2.1–14.0)
Gas	0.235	(0.164–0.336)	−76.5	(−83.6–−66.4)
Drowning	0.775	(0.663–0.905)	−22.5	(−33.7–−9.5)
Poisoning	2.750	(2.288–3.304)	175.0	(128.8–230.4)
Others	1.456	(1.292–1.642)	45.6	(29.2–64.2)
**Age 80+**				
Hanging	1.517	(1.394–1.650)	51.7	(39.4–65.0)
Gas	0.191	(0.060–0.608)	−80.9	(−94.0–−39.2)
Drowning	1.195	(0.920–1.552)	19.5	(−8.0–55.2)
Poisoning	3.494	(2.552–4.783)	249.4	(155.2–378.3)
Others	1.376	(1.056–1.794)	37.6	(5.6–79.4)

Note. IRR = Incidence rate ratio; CI = Confidence Interval.

## Supplementary Materials

The following are available online at https://www.mdpi.com/1660-4601/16/10/1794/s1, Figure S1-1: Trends in age-standardized and crude suicide mortality by method, 1979–2016 for men, Figure S1-2: Trends in age-standardized and crude suicide mortality by method, 1979–2016 for women, Figure S2-1: Trends in suicide mortality by age group, 1979–2016 among men, Figure S2-2: Trends in suicide mortality by age group, 1979–2016 among women, Table S1: ICD codes used for suicide definition, by year and ICD era, Table S2: Poisson regression analysis of suicide by age and suicide categories, 1979–2016, by sex.
