# Supplementary material for: Trends in Suicide Mortality by Method from 1979 to 2016 in Japan
Source: Int J Environ Res Public Health. 2019 May 21;16(10):1794. doi: 10.3390/ijerph16101794 (PMC6571574; doi:10.3390/ijerph16101794)
Supplement: Supplementary file 1 [file ijerph-16-01794-s001.pdf]

Supplementary files

Table of Contents

1. Supplementary Methods 2

Data sources 2

Detail of model equations 2

Base models 5

2. Supplementary results 5

List of tables

Table S1: ICD codes used for suicide definition, by year and ICD era 2

Table S2: Poisson regression analysis of suicide by age and suicide categories, 1979–2016, by sex 11

List of figures

Figure S1-1: Trends in age-standardized and crude suicide mortality by method, 1979–2016 for men 6

Figure S1-2: Trends in age-standardized and crude suicide mortality by method, 1979–2016 for women 8

8

Figure S2-1 Trends in suicide mortality by age group, 1979–2016 among men 9

Figure S2-2 Trends in suicide mortality by age group, 1979–2016 among women 10

1. Supplementary Methods

Data sources

Table S1 shows the International Classification of Disease (ICD) codes used to define suicide categories by ICD era.

Table S1: ICD codes used for suicide definition, by year and ICD era.

Years	ICD version	Overall ICD codes	ICD codes by method				
			Drowning	Gas	Hanging	Poisoning	Other
1979–1994	9	E950–E959	E954	E951, E952	E953	E950	E955, E956, E957, E958
1995–2016	10	X60–X84	X71	X67	X70	X60–X66, X68–X69	X72–X83

Detail of model equations

Data are modeled as Poisson distributed, with population included in the model as an offset to ensure that changes in population structure are accounted for. The model is conducted separately by sex and includes a term for a linear time trend, with interaction terms to allow different time trends by age category and method of suicide. A step term (0 before 1998, 1 after) is included to model the impact of the 1998 increase in suicide rates. To answer the key research question, a four-way interaction between suicide type, the

step term, age category, and time was tested. This four-way interaction, if significant, indicates that the effect of the 1998 step on trend in suicide differed by age and suicide category.

This can be written in equation form for the simplest case as follows. Suppose that at time i we have data on the number of suicide deaths y_i, occurring at rate μ_i in population n_i.

Then, we can describe the fundamental distribution of the data as:

$$y_i \sim Poisson(\mu_i)$$

where the rate μ_i is related to the covariates through a log-linear expression as follows:

$$\begin{aligned}\ln(\mu_i) = \alpha + \ln(n_i) + \beta_1 x_{i1} + \beta_2 x_{i2} + \beta_3 x_{i3} + \beta_4 x_{i4} + \beta_5 x_{i1} x_{i2} \\ + \beta_6 x_{i1} x_{i3} + \beta_7 x_{i1} x_{i4} + \beta_8 x_{i2} x_{i3} + \beta_9 x_{i2} x_{i4} + \beta_{10} x_{i3} x_{i4} + \beta_{11} x_{i1} x_{i2} x_{i3} \\ + \beta_{12} x_{i1} x_{i2} x_{i4} + \beta_{13} x_{i1} x_{i3} x_{i4} + \beta_{14} x_{i2} x_{i3} x_{i4} + \beta_{15} x_{i1} x_{i2} x_{i3} x_{i4}\end{aligned}$$

Where,

α is the intercept term
x_{i1} is the year, with the first year in the data series (e.g. 1979) set to be 0
x_{i2} is the suicide category (1 for hanging, 2 for gas, 3 for drowning, 4 for poisoning, and 5 for other methods)
x_{i3} is the age category (1 for 15–29, 2 for 30–59, 3 for 60–79, and 4 for 80+)
x_{i4} is the step function (0 for years <1998, 1 for 1998, and onward)

In this equation, all the categorical variables with more than two levels (age group and method) are actually composed as sets of dummy variables, so in fact, x_2 is composed of a set of four dummy variables. However, for simplicity in the model equation, we have written these sets of dummy variables as a single term. Where interactions are depicted, all the dummy variables are entered into the interaction as a single group and removed together, which is in accordance with standard statistical practice. In this case, the coefficients of these variables (e.g. β_5) should be interpreted as actually reflecting a set of coefficients attached to all the combinations of dummy variables in the interaction term.

The coefficients in this model measure the following effects:

β_1 measures the time trend in hanging-related mortality for people aged 15–29 before the 1998 increase
β_2 measures the rate ratio of mortality among suicide methods for people aged 15–29 at the starting year
β_3 measures the rate ratio of mortality among various age categories who committed suicide before 1998 by hanging
β_4 measures the sudden increase or decrease in suicide mortality in 1998 relative to 1997
β_5 measures the difference in trend for various suicide methods among people aged 15–29 in the period before 1998
β_6 measures the trend in hanging mortality by age categories before 1998
β_7 measures the change in trend in hanging among people aged 15–29 in 1998
β_8 measures the rate ratio of mortality by various suicide methods in different age categories before 1998
β_9 measures the sudden increase or decrease in suicide rates in 1998 relative to 1997 for hanging suicide among people aged 15–29 (the additional impact of the 1998 step on the level of suicide rates by various methods)
β_{10} measures the change in step in 1998 by age category for hanging suicide
β_{11} measures the change in trend before 1998 by age and suicide category

β_{12} measures the change in trend in various suicide methods among 15–29 aged people in 1998

β_{13} measures the change in hanging trend in various age categories in 1998

β_{14} measures the increase in step in 1998 by age and suicide categories

β_{15} measures the additional change in trend in suicide by various age and suicide categories in 1998

The study estimates these key parameters for suicide, separately by sex, adjusting for broad age groups and categorizing by age group and methods of suicide. The various categories of suicide used are poisonings, drownings, gas, hanging and other, while the age was divided into 15–29, 30–59, 60–79, and 80+. The results for all the suicide categories are presented as supplementary results.

Base models

There are two basic models for this study:

- Suicide mortality by age and suicide category
- Proportion of hanging suicide

Both the analyses are modeled separately by sex, for data from 1979–2016, with the 1998 increase in suicide rates.

2. Supplementary results

This section shows additional information on mortality by suicide categories.

Figure S1-1 and S1-2 show the trends in age standardized and crude suicide mortality by the top three methods for men and women. These figures clearly show that in all years

from 1979 until 2016, hanging was the most commonly used method to commit suicide among both men and women. Suicide by hanging was higher in men compared to women. Hanging was followed by gas and poisoning in men and by poisoning and drowning in women. Both the figures show that the rates of drowning, poisoning, and gas did not increase sharply in 1998, leaving hanging as the main method of suicide. In men (Figure S1-1), suicide by poisoning increased during the mid-1980s, after which it leveled at very low rates until 2016. Suicide by gassing among men, which did not increase much around 1998, instantaneously became more common among men after 2002. The age-standardized suicide rate for drowning among women (Figure S1-2) was declining steeply throughout the period studied compared to the crude rate. Suicide by poisoning and drowning among women, which was often high, suddenly plateaued after 1998.

Figure S1-1: Trends in age-standardized and crude suicide mortality by method, 1979–2016 for men

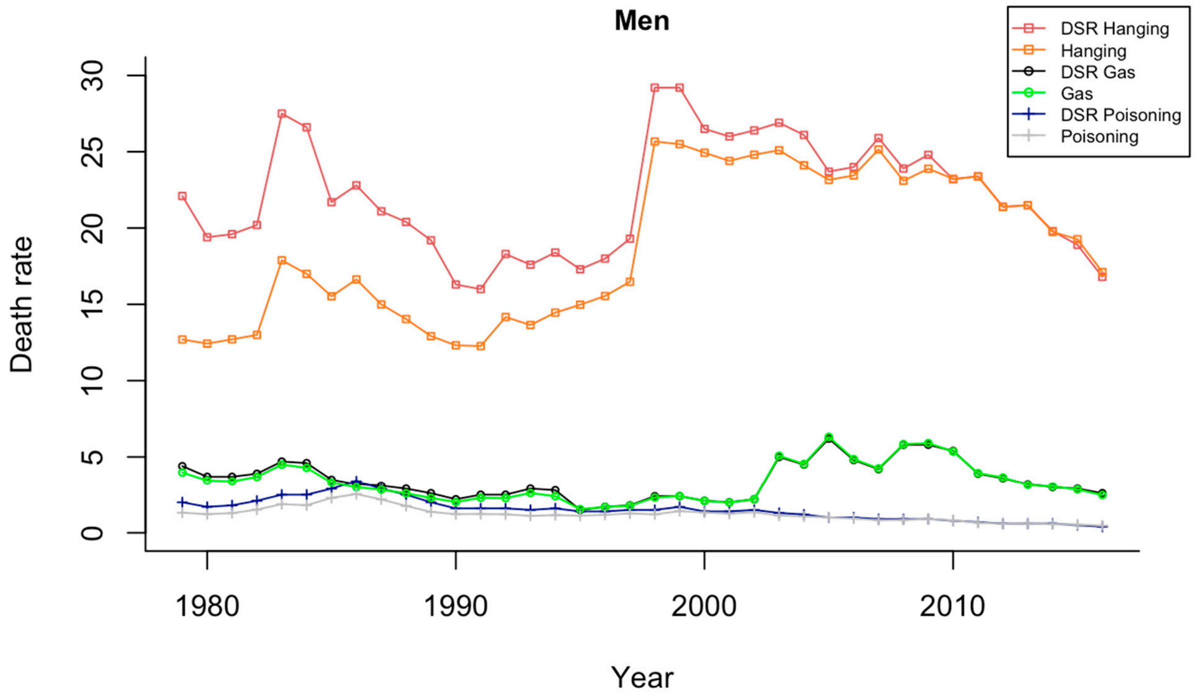

Men
DSR Hanging
Hanging
DSR Gas
Gas
DSR Poisoning
Poisoning
Death rate
0
5
10
15
20
25
30
1980
1990
2000
2010
Year

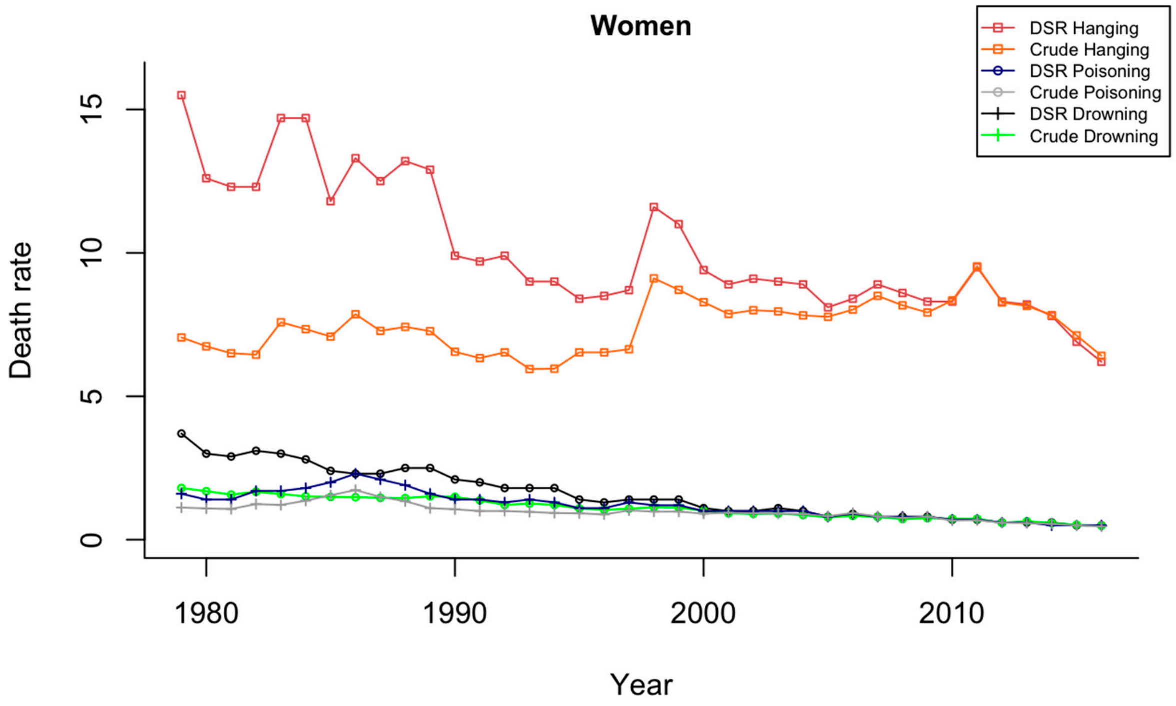

Figure S1-2: Trends in age-standardized and crude suicide mortality by method, 1979–2016 for women

Figure S2-1 and S2-2 show the trends in suicide rates by age category for men and women from 1979 to 2016. The suicide trend for people aged 80+ follows a broad downward trend in both sexes. However, among men aged 30–79, suicide rates increased slightly after 1997 (Figure S2-1). These figures clearly show that suicide rates increase with any increase in age, but are decreasing rapidly in the groups with the highest rates. Note that even in the narrower age categories shown here, the effect of aging within the narrower age categories may lead to an appearance of stable rates when they are actually mildly

decreasing. Since plots of five-year age groups are too complex to depict, age-specific rates in very narrow categories are not shown.

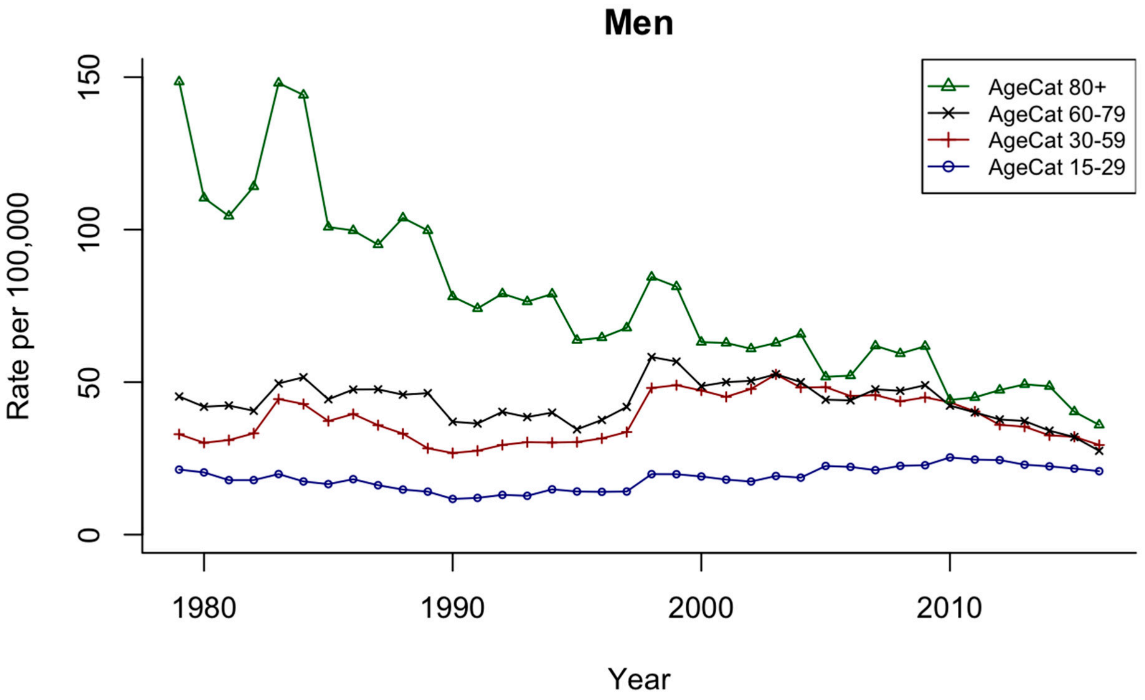

Figure S2-1 Trends in suicide mortality by age group, 1979–2016 among men

Table S2 shows the full result of the Poisson regression analysis of suicide by age and

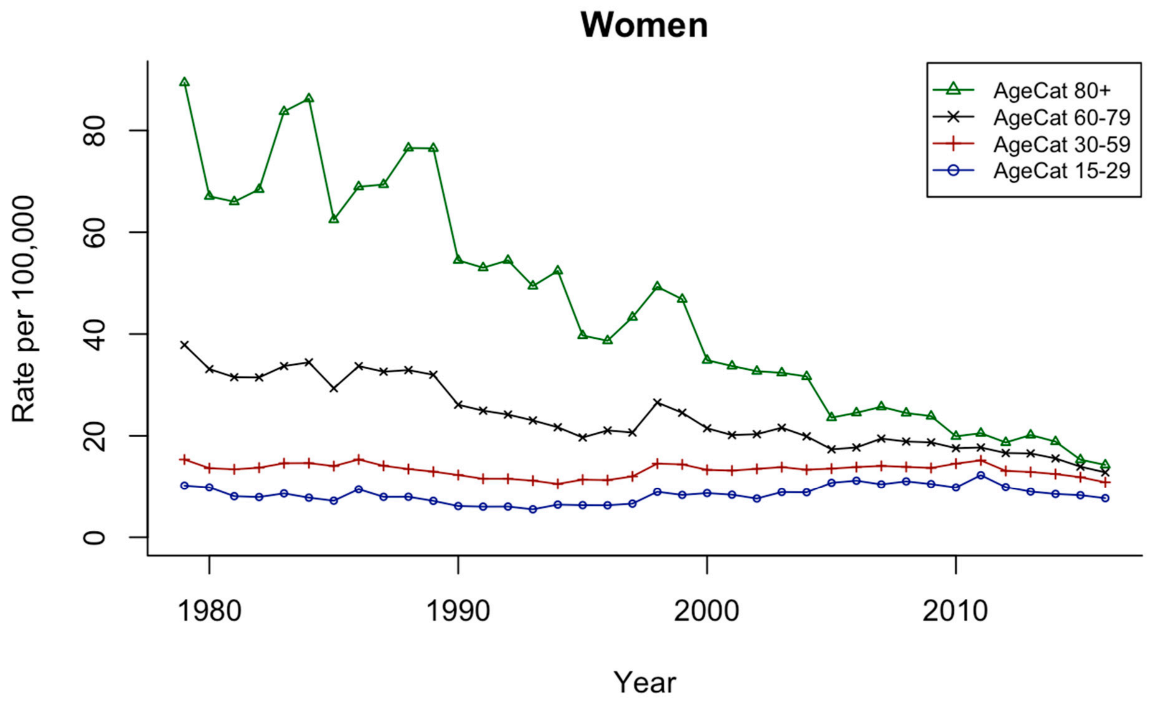

Figure S2-2 Trends in suicide mortality by age group, 1979–2016 among

suicide categories, separately by sex. For simplicity, the reference categories in the interaction terms are collapsed to a single category (labeled "Reference levels") to avoid complexity in the table. This shows that suicide rates increased with increase in age. Moreover, mortality due to hanging was particularly high in the older age groups.

In this table, the four-way interaction for men shows that gas and poisoning showed a reduction in trend after 1998 that was even greater than the reduction in the trend in hanging. The step/age category interaction shows the very large increase in hanging in older men (aged 30–79) after 1998 compared to before. This is the key driver of the size of the step after 1998.

Table S2: Poisson regression analysis of suicide by age and suicide categories, 1979–2016, by sex.

	Men		
**Variable**	**IRR**	**P-value**	**95% CI**
**Year**	0.989	0.000	0.986–0.991
**Suicide Category**			
Hanging	Ref		
Gas	0.616	0.000	0.586–0.647
Drowning	0.090	0.000	0.081–0.099
Poisoning	0.123	0.000	0.113–0.135
Other	0.700	0.000	0.671–0.730
**Suicide Category / Year Interaction**			
Hanging	Ref		
Gas	0.911	0.000	0.906–0.917
Drowning	0.972	0.000	0.962–0.982
Poisoning	0.961	0.000	0.952–0.970
Other	0.999	0.591	0.995–1.003
**Age Category**			
Age 15–29	Ref		
Age 30–59	2.356	0.000	2.286–2.428
Age 60–79	4.221	0.000	4.082–4.364
Age 80+	13.012	0.000	12.433–13.618
**Age Category / Year interaction**			
Age 15–29	Ref		
Age 30–59	1.010	0.000	1.007–1.013
Age 60–79	0.998	0.202	0.995–1.001
Age 80+	0.972	0.000	0.968–0.976
**Suicide Category / Age Category interaction**			
Reference levels	Ref		
Gas / age 30–59	0.489	0.000	0.462–0.518
Gas / age 60–79	0.060	0.000	0.054–0.067
Gas / age 80+	0.025	0.000	0.018–0.036
Drowning / age 30–59	0.828	0.001	0.741–0.926
Drowning / age 60–79	0.721	0.000	0.634–0.820
Drowning / age 80+	0.882	0.165	0.739–1.053
Poisoning / age 30–59	1.236	0.000	1.123–1.359
Poisoning / age 60–79	0.903	0.062	0.811–1.005
Poisoning / age 80+	0.411	0.000	0.343–0.493

Other / age 30–59	0.554	0.000	0.527–0.581
Other / age 60–79	0.243	0.000	0.227–0.259
Other / age 80+	0.247	0.000	0.222–0.275
**Suicide Category / Age Category / Year interaction**			
Reference levels	Ref		
Gas / age 30–59	1.052	0.000	1.045A–1.058
Gas / age 60–79	1.109	0.000	1.098–1.121
Gas / age 80+	1.023	0.230	0.986–1.062
Drowning / age 30–59	1.018	0.002	1.007–1.030
Drowning / age 60–79	1.010	0.136	0.997–1.023
Drowning / age 80+	0.986	0.121	0.968–1.004
Poisoning / age 30–59	1.003	0.541	0.993–1.013
Poisoning / age 60–79	1.039	0.000	1.028–1.050
Poisoning / age 80+	1.065	0.000	1.047–1.083
Other / age 30–59	0.996	0.095	0.991–1.001
Other / age 60–79	1.016	0.000	1.010–1.022
Other / age 80+	0.985	0.004	0.975–0.995
**1998 Step**	1.127	0.000	1.054–1.204
**Step / Year interaction**			
Before 1998	Ref		
After 1998	1.024	0.000	1.021 -1.027
**Suicide Category / Step interaction**			
Hanging / After 1998	Ref		
Gas / After 1998	0.074	0.000	0.063–0.086
Drowning / After 1998	0.618	0.019	0.413–0.924
Poisoning / After 1998	1.524	0.009	1.108–2.094
Other / After 1998	1.133	0.053	0.999–1.285
**Suicide Category / 1998 Step / Year interaction**			
Hanging / After 1998	Ref		
Gas / After 1998	1.165	0.000	1.156–1.174
Drowning / After 1998	0.997	0.773	0.980–1.015
Poisoning / After 1998	0.981	0.012	0.967–0.996
Other / After 1998	0.970	0.000	0.964–0.976
**Age Category / 1998 Step interaction**			
Age 15–29 / After 1998	Ref		
Age 30–59 / After 1998	2.688	0.000	2.500–2.890
Age 60–79 / After 1998	2.004	0.000	1.854–2.166

Age 80+ / After 1998	0.978	0.682	0.878 -1.089
**Age Category / 1998 Step / Year interaction**			
Age 15–29 / After 1998	Ref		
Age 30–59 / After 1998	0.954	0.000	0.950–0.957
Age 60–79 / After 1998	0.961	0.000	0.957–0.965
Age 80+ / After 1998	0.983	0.000	0.978–0.988
**Suicide Category / Age Category / 1998 Step interaction**			
Reference levels	Ref		
Gas / age 30–59 / After 1998	4.292	0.000	3.632–5.073
Gas / age 60–79 / After 1998	17.655	0.000	14.022–22.229
Gas / age 80+ / After 1998	2.595	0.027	1.114–6.046
Drowning / age 30–59 / After 1998	1.870	0.005	1.209–2.893
Drowning / age 60–79 / After 1998	0.938	0.784	0.595–1.480
Drowning / age 80+ / After 1998	0.860	0.636	0.462–1.603
Poisoning / age 30–59 / After 1998	0.401	0.000	0.283–0.568
Poisoning / age 60–79 / After 1998	1.225	0.280	0.848–1.770
Poisoning / age 80+ / After 1998	1.409	0.169	0.864–2.298
Other / age 30–59 / After 1998	0.692	0.000	0.600–0.799
Other / age 60–79 / After 1998	0.966	0.684	0.819–1.140
Other / age 80+ / After 1998	0.781	0.090	0.587–1.039
**Suicide Category / Age Category / 1998 Step / Year interaction**			
Reference levels	Ref		
Gas / age 30–59 / After 1998	0.924	0.000	0.916–0.932
Gas / age 60–79 / After 1998	0.861	0.000	0.850–0.872
Gas / age 80+ / After 1998	0.966	0.135	0.923–1.011
Drowning / age 30–59 / After 1998	0.971	0.003	0.953–0.990
Drowning / age 60–79 / After 1998	1.019	0.078	0.998–1.040
Drowning / age 80+ / After 1998	1.033	0.021	1.005–1.063
Poisoning / age 30–59 / After 1998	1.022	0.008	1.006–1.039
Poisoning / age 60–79 / After 1998	0.962	0.000	0.945–0.978
Poisoning / age 80+ / After 1998	0.970	0.012	0.948–0.994
Other / age 30–59 / After 1998	1.022	0.000	1.016–1.029
Other / age 60–79 / After 1998	1.011	0.009	1.003–1.019
Other / age 80+ / After 1998	1.037	0.000	1.023–1.052
**_cons**	0.000	0.000	0.000–0.000
**offS**	1 (offset)		
	**Women**		
**Variables**	**IRR**	**P-value**	**95% CI**

**Year**	0.989	0.000	0.984–0.993
**Suicide Category**			
Hanging	Ref		
Gas	0.896	0.009	0.826–0.973
Drowning	0.300	0.000	0.269–0.336
Poisoning	0.346	0.000	0.312–0.383
Other	1.373	0.000	1.287–1.465
**Suicide Category / Year interaction**			
Hanging	Ref		
Gas	0.875	0.000	0.866–0.885
Drowning	0.940	0.000	0.928–0.951
Poisoning	0.961	0.000	0.950–0.971
Other	1.008	0.011	1.002–1.014
**Age Category**			
Age 15–29	Ref		
Age 30–59	2.651	0.000	2.512–2.798
Age 60–79	9.864	0.000	9.348–10.408
Age 80+	25.381	0.000	23.889–26.966
**Age Category / Year interaction**			
Age 15–29	Ref		
Age 30–59	0.999	0.792	0.994–1.005
Age 60–79	0.977	0.000	0.972–0.982
Age 80+	0.973	0.000	0.968–0.979
**Suicide Category / Age Category interaction**			
Reference levels	Ref		
Gas / age 30–59	0.281	0.000	0.255–0.310
Gas / age 60–79	0.044	0.000	0.038–0.052
Gas / age 80+	0.012	0.000	0.008–0.018
Drowning / age 30–59	0.894	0.072	0.791–1.010
Drowning / age 60–79	0.743	0.000	0.657–0.841
Drowning / age 80+	0.747	0.000	0.649–0.860
Poisoning / age 30–59	0.823	0.001	0.734–0.922
Poisoning / age 60–79	0.364	0.000	0.323–0.410
Poisoning / age 80+	0.140	0.000	0.117–0.168
Other / age 30–59	0.364	0.000	0.338–0.392
Other / age 60–79	0.110	0.000	0.101–0.120
Other / age 80+	0.068	0.000	0.059–0.078
**Suicide Category / Age Category / Year interaction**			
Reference levels	Ref		

Gas / age 30–59	1.047	0.000	1.034–1.060
Gas / age 60–79	1.045	0.000	1.026–1.065
Gas / age 80+	1.049	0.040	1.002–1.099
Drowning / age 30–59	1.051	0.000	1.037–1.065
Drowning / age 60–79	1.046	0.000	1.032 1.060
Drowning / age 80+	1.041	0.000	1.025–1.056
Poisoning / age 30–59	1.010	0.105	0.998–1.022
Poisoning / age 60–79	1.060	0.000	1.047–1.073
Poisoning / age 80+	1.086	0.000	1.068–1.104
Other / age 30–59	1.009	0.013	1.002–1.016
Other / age 60–79	1.021	0.000	1.013–1.029
Other / age 80+	0.999	0.849	0.987–1.011
**1998 step**	1.243	0.000	1.111–1.391
**1998 Step / Year interaction**			
Before 1998	Ref		
After 1998	1.027	0.000	1.021–1.033
**Suicide Category / 1998 Step interaction**			
Hanging / After 1998	Ref		
Gas / After 1998	0.028	0.000	0.021–0.038
Drowning / After 1998	0.481	0.003	0.296–0.783
Poisoning / After 1998	0.771	0.090	0.571–1.041
Other / After 1998	1.490	0.000	1.251–1.775
**Suicide Category / 1998 Step / Year interaction**			
Hanging / After 1998	Ref		
Gas / After 1998	1.223	0.000	1.206–1.240
Drowning / After 1998	1.020	0.070	0.998–1.042
Poisoning / After 1998	1.017	0.023	1.002–1.033
Other / After 1998	0.948	0.000	0.940–0.956
**Age Category / 1998 Step interaction**			
Age 15–29 / After 1998	Ref		
Age 30–59 / After 1998	0.899	0.097	0.792–1.020
Age 60–79 / After 1998	0.860	0.020	0.758–0.976
Age 80+ / After 1998	1.243	0.003	1.077–1.434
**Age Category / 1998 Step / Year interaction**			
Age 15–29 / After 1998	Ref		
Age 30–59 / After 1998	0.986	0.000	0.980–0.993
Age 60–79 / After 1998	0.983	0.000	0.976–0.989
Age 80+ / After 1998	0.956	0.000	0.949–0.963

**Suicide Category / Age Category / 1998 Step interaction**			
Reference levels	Ref		
Gas / age 30–59 / After 1998	6.526	0.000	4.647–9.165
Gas / age 60–79 / After 1998	6.715	0.000	4.160–10.838
Gas / age 80+ / After 1998	3.842	0.034	1.108–13.324
Drowning / age 30–59 / After 1998	4.517	0.000	2.685–7.599
Drowning / age 60–79 / After 1998	1.482	0.135	0.884–2.485
Drowning / age 80+ / After 1998	1.639	0.086	0.932–2.885
Poisoning / age 30–59 / After 1998	0.953	0.787	0.673–1.350
Poisoning / age 60–79 / After 1998	3.549	0.000	2.474–5.092
Poisoning / age 80+ / After 1998	3.172	0.000	2.016–4.993
Other / age 30–59 / After 1998	1.077	0.471	0.880–1.319
Other / age 60–79 / After 1998	0.930	0.522	0.744–1.162
Other / age 80+ / After 1998	0.609	0.004	0.434–0.854
**Suicide Category / Age Category / 1998 Step / Year interaction**			
Reference levels	Ref		
Gas / age 30–59 / After 1998	0.926	0.000	0.911–0.942
Gas / age 60–79 / After 1998	0.934	0.000	0.911–0.956
Gas / age 80+ / After 1998	0.945	0.063	0.889–1.003
Drowning / age 30–59 / After 1998	0.928	0.000	0.906–0.949
Drowning / age 60–79 / After 1998	0.987	0.256	0.965–1.010
Drowning / age 80+ / After 1998	0.980	0.106	0.955–1.004
Poisoning / age 30–59 / After 1998	0.988	0.157	0.971–1.005
Poisoning / age 60–79 / After 1998	0.915	0.000	0.899–0.931
Poisoning / age 80+ / After 1998	0.926	0.000	0.905–0.947
Other / age 30–59 / After 1998	1.015	0.004	1.005–1.025
Other / age 60–79 / After 1998	1.027	0.000	1.016–1.038
Other / age 80+ / After 1998	1.055	0.000	1.038–1.073
**Constant***	0.000	0.000	0.000–0.000
**Population offset**	1 (offset)		

Note. IRR = Incidence rate Ratio; CI = Confidence Interval

*The constant in this model is the rate in 15–29 year olds, in 1979, who died by hanging. This rate is less than 1 per 1000, and so is 0 to three decimal places in these tables.
